# Cellular Response to Ionizing Radiation: A MicroRNA Story

**Published:** 2012

**Authors:** Mohammad Halimi, S. Mohsen Asghari, Reyhaneh Sariri, Dariush Moslemi, Hadi Parsian

**Affiliations:** 1 *Department of Biology, Faculty of Sciences, University of Guilan, Rasht, Iran.*; 2 *Department of Radiation oncology, Babol University of Medical Sciences, Babol, Iran.*; 3 *Cellular and Molecular Biology Research Center, Babol University of Medical Sciences, Babol, Iran.*; 4 *Department of Biochemistry and Biophysics, Babol University of Medical Sciences, Babol, Iran.*

**Keywords:** Cellular response, ionizing radiation, microRNA

## Abstract

MicroRNAs (miRNAs) represent a class of small non-coding RNA molecules that regulate gene expression at the post-transcriptional level. They play a crucial role in diverse cellular pathways. Ionizing radiation (IR) is one of the most important treatment protocols for patients that suffer from cancer and affects directly or indirectly cellular integration. Recently it has been discovered that microRNA-mediated gene regulation interferes with radio-related pathways in ionizing radiation. Here, we review the recent discoveries about miRNAs in cellular response to IR. Thoroughly understanding the mechanism of miRNAs in radiation response, it will be possible to design new strategies for improving radiotherapy efficiency and ultimately cancer treatment.

Ionizing radiation (IR) is widely used for the treatment of cancer. IR directly damage cellular components or generate reactive oxygen (ROS) and nitrogen (RNS) species that can disrupt atomic structure of macromolecules (1). DNA is the primary target for cell damage from IR (2). There are two major types of IR including photons (x-rays and γ-rays), which are most widely used in radiotherapy and particle radiation (electrons, protons, neutrons, carbon ions, α-particles, and β-particles). In X and γ-rays, most of the radiation effects are through free radicals whereas in particle radiation, direct damage of DNA is more important. In this review, the word "ionizing radiation" refers to all these radiations and their effects. A series of signaling pathways begin in cells following exposure to IR that may arrest the cell cycle and repair the damage or induce apoptosis if damage is extensive or in some cases enhance cell division and resistance to cytotoxic stresses (3).

MicroRNAs (miRNAs) are a newly discovered class of non-coding and endogenous RNA molecules that regulate gene expression at the post-transcriptional level (4). These single-strand RNA molecules are about 18-22 nucleotides long that incorporate and act in a nuclear protein complex named RNA induced silencing complex (RISC). In this complex miRNAs interact with 3′ untranslated region (3′ UTR) of target mRNAs at specific sites (5, 6). If complete complementation occurs between the miRNA and target mRNA, Argonaute 2 (Ago2) (an RNase enzyme), cleaves the mRNA, otherwise translation is repressed without degradation of target mRNA (7-9). So far, about 1500 miRNAs have been identified in human. A single miRNA can bind many mRNAs and multiple miRNAs can target a single mRNA. Therefore, there is a complex regulatory network between miRNAs and their target genes (10, 11).

IR triggers several biochemical and signaling events and alters expression of many genes involved in cell cycle regulation, checkpoints, apoptosis, and signal transduction pathways (12). Since miRNAs participate in control of an estimated >30% of all human genes, it is logical that miRNAs be also involved in the regulation of IR-induced gene expression. In this review, we illustrate the detailed regulatory mechanisms of miRNAs in modulating cellular response to ionizing radiation from diverse aspects.

**IR-induced DNA damage response** **(DDR)**

The main toxic biological effect of IR is DNA single (SSB) or double (DSB) strand break. Cells are very sensitive to DNA breaks especially at late G2, and during M phase (13, 14). Several genes upregulate after DNA break. The product of these genes could be categorized in three groups including sensors, transducers, and effectors. The final effect of all these processes is cell cycle arrest and DNA repair, or if the damage is extensive, apoptosis will occur (15). The first group of molecules that detect DNA breaks are sensors. The major known sensors are Mre11/Rad50/NBS1 (MRN)-complex and ataxia telangiectasia mutated (ATM). The main known transducers are Mouse double minute 2 homolog (MDM2), p53, Silent information regulator 1 (SIRT1), Checkpoint kinase 2 (Chk2), breast cancer type 1 susceptibility protein (BRCA1), and finally the major effectors are Bcl-2–associated X protein (BAX), cyclin-dependent kinase inhibitor 1 (p21), Cdk2/Cycline E, Rad51 and Rad50/Mre11 (-). Recently with discovery of miRNAs, a new layer of complexity has been added to IR signaling (19).

**Role of miRNAs in IR-induced DDR**

Following double-strand breaks by IR, the MRN complex binds to broken ends of DNA. MRN complex recruits ATM to broken DNA molecules and fully activates ATM (20). ATM, in its active form, phosphorylates several key proteins that initiate activation of the DNA damage checkpoint, leading to cell cycle arrest, DNA repair or apoptosis (21, 22). Hu et al. showed that miR-421, suppresses ATM expression by targeting the 3′ UTR of ATM transcripts. They also observed that ectopic expression of miR-421 results in S-phase cell cycle checkpoint changes and an increased sensitivity to ionizing radiation, creating a cellular phenotype similar to that of cells derived from ataxia-telangiectasia (A-T) patients (23). MiR-101 can efficiently target ATM via binding to the 3^′^ UTR of ATM mRNA and upregulated miR-101 decreases the protein levels of ATM. Upregulating miR-101, sensitizes the tumor cells to radiation in vitro and in vivo. MiRNA biogenesis is globally induced upon DNA damage in an ATM-dependent manner (24). About one fourth of miRNAs are significantly upregulated after DNA damage, while loss of ATM abolishes their induction. ATM phosphorylates KH-type splicing regulatory protein (KSRP), a key player of miRNA biogenesis, and increases its activity and thereby miRNA processing (Fig. 1) (25). 

Active ATM in its monomeric form has about 700 substrates that one of them is p53. ATM phosphorylates p53 that leads to its dissociation from MDM-2, an inhibitor of p53, and activation of p53 (27). p53 in its active form phosporylates and activates p21. Activated p21 binds to and inactivates CDK-Cyclin complexes and thereby arrests cell cycle in G1→S, S phase and G2→M. p53 induces apoptosis through transcriptional activation of several pro-apoptotic factors (28). However, there are newly discovered pathways mediated by some miRNAs. Activated p53 induces the expression of miR-34a/b/c (-). miR-34a/b/c downregulate CDK4 and CDK6 to induce cell cycle arrest and downregulate BCL2 to promote apoptosis (34). p53 also enhances expression of miR-192 and miR-215. These miRNAs, downregulate transcription of CDC7, MAD2L1 and CUL5, that are involved in cell proliferation (Fig. 2) (35, 36).

Several miRNAs directly or indirectly effect p53 expression. Hu et al. reported that miR-504 acts as a negative regulator of human p53 through its direct binding to two sites in p53 3′ UTR. They reported that overexpression of miR-504 decreases p53 protein levels and functions in cells, including p53 transcriptional activity, p53-mediated apoptosis and cell cycle arrest in response to stress (37). Le et al. demonstrated that miR-125b, is a negative regulator of p53 in both zebrafish and humans. They found that miR-125b-mediated downregulation ability of p53, is strictly dependent on the binding of miR-125b to a microRNA response element in the 3^′ ^UTR of p53 mRNA. They also observed that knockdown of miR-125b elevates the level of p53 protein and induces apoptosis in human lung fibroblasts and in the zebrafish brain. Interestingly, miR-125b was downregulated when zebrafish embryos are treated with γ-irradiation, corresponding to the rapid increase in p53 protein in response to DNA damage. Ectopic expression of miR-125b suppressed the increase of p53 and stress-induced apoptosis (38). 

MDM2 is a direct inhibitor of p53. There is an inverse correlation between miR-221 and Mdm2 protein levels. MiR-221 directly regulates Mdm2 expression and increases p53 protein levels and stability (39). Both miR-143 and miR-145, which belong to the same miRNA cluster, target the miRNA regulatory elements (MREs) in the 3′ UTR of MDM2. These molecules are post-transcriptionally activated by p53, thereby generating a short miRNAs-MDM2-p53 feedback loop (Fig 3) (40). Silent information regulator 1 (SIRT1) is a negative regulator of p53. MiR-132 was shown to inhibit SIRT1 expression through a miR-132 binding site in the 3′ UTR of SIRT1 (41). Yamakuchi et al. showed that MiR-34a inhibits SIRT1 expression through a miR-34a-binding site within the 3′ UTR of SIRT1 (42).

**Fig 1:**
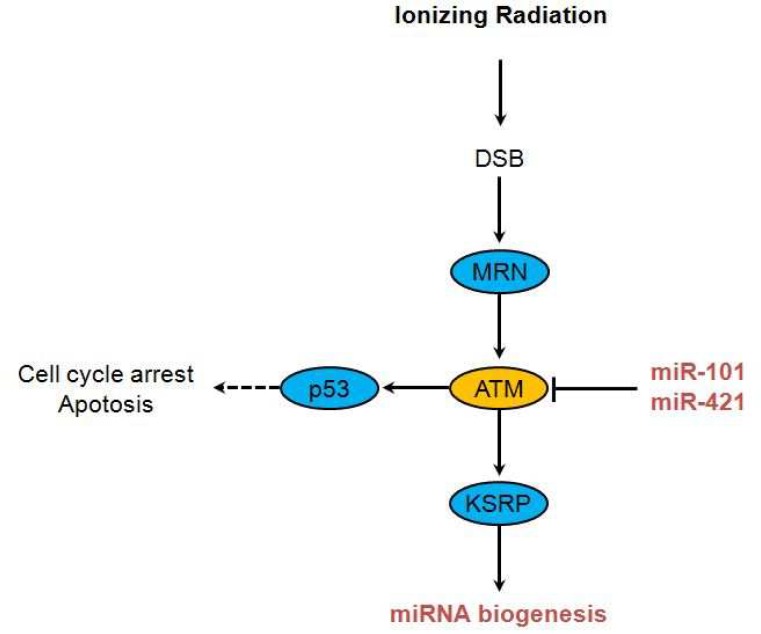
ATM associated miRNAs. After ionizing radiation and DSB, MRN complex binds to broken ends of DNA, then recruits and activates ATM. MiR-101 and miR-421 are suppressors of ATM expression. This information is resulted from references (21-26)

**Fig 2:**
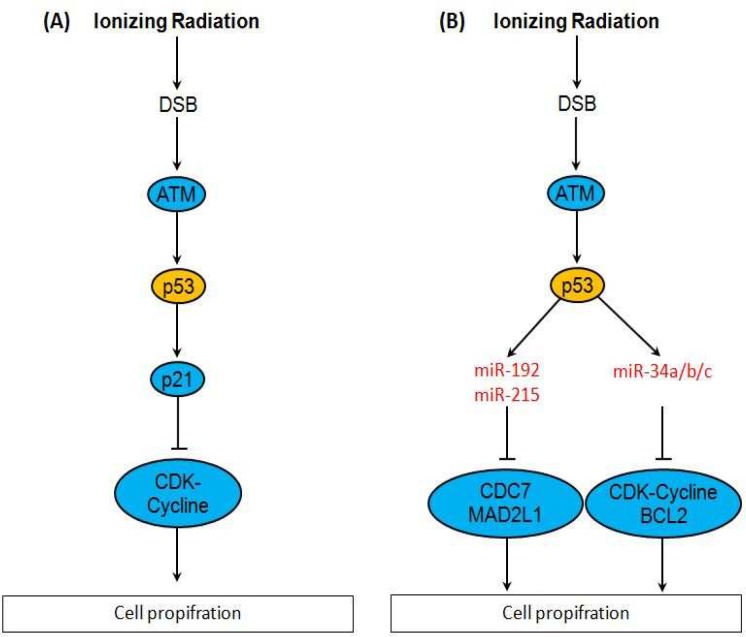
DSB activates p21 that inhibits cell proliferation by inactivating CDK-Cycline complexes **(A)**. p53 induces miR-34a/b/c, miR-192 and miR-215 transcription. These miRNAs inhibit cell proliferation by downregulating CDC2, MAD2L1, CDK-Cycline and BCL2 **(B)** (27-36)

**MiRNAs and radiation induced signal trans-duction pathways**

IR not only causes cell death, but also has potential of enhancing proliferation in the surviving fraction of cells (43, 44). IR damages DNA and generates ROS that the later products in addition to activation of p53 and ATM, are able to activate growth factor receptors in the plasma membrane (44). Protein tyrosine phosphatases (PTPases) are key regulatory components in signal transduction pathways that regulate the phosphorylation state of many important signaling molecules. PTPases dephosphorylate and activate several intermediates of signal transduction pathways. ROS is an inhibitor of PTPases. Therefore, radiation decreases PTPases activities by generating ROS and consequently increased phosphoryation and activity of several proteins especially receptor tyrosin kinases will occur (45, 46). 

Recent evidences suggested that microRNA-mediated gene regulation interconnects with the radio-related signal transduction pathways. Webster et al. in a study on several cancer cell lines (lung, breast, and glioblastoma) found that miR-7 downregulates EGFR mRNA and protein expression via two sites on 3^' ^UTR of EGFR mRNA, inducing cell cycle arrest and cell death (47). Furthermore in another study, researchers showed that ectopic over expression of miR-7, attenuated EGFR and Protein Kinase B (Akt) expression and radio-sensitized MDA-MB-468 breast cancer cells (48). p85β is a regulatory subunit involved in stabilizing and propagating the PI3K signal. p85β 3^'^ UTR is directly targeted by miR-126 (49). Phosphoinositide 3-kinase catalytic subunit delta (PIK3CD) and p70S6K are also miR-7 targets (50).

 The seed sequence of miR-221 and miR-222 matched the 3' UTR of Phosphatase and tensin homolog (PTEN) mRNA, and PTEN-3' UTR luciferase reporter assay confirmed PTEN as a direct target of miR-221 and miR-222 (51). MiR-26a and miR-21 are direct regulators of PTEN expression (52, 53). Transcription of miR-486 is directly controlled by Serum Response Factor (SRF), Myocardin-related transcription factor A (MRTF-A) and MyoD. PTEN and Forkhead box protein O1A (Foxo1a) are targets of miR-486, which negatively affect phosphoinositide-3-kinase (PI3K)/Akt signaling. MRTF-A promotes PI3K/Akt signaling by upregulating miR-486 expression, (Fig4) (54).

**Fig 3:**
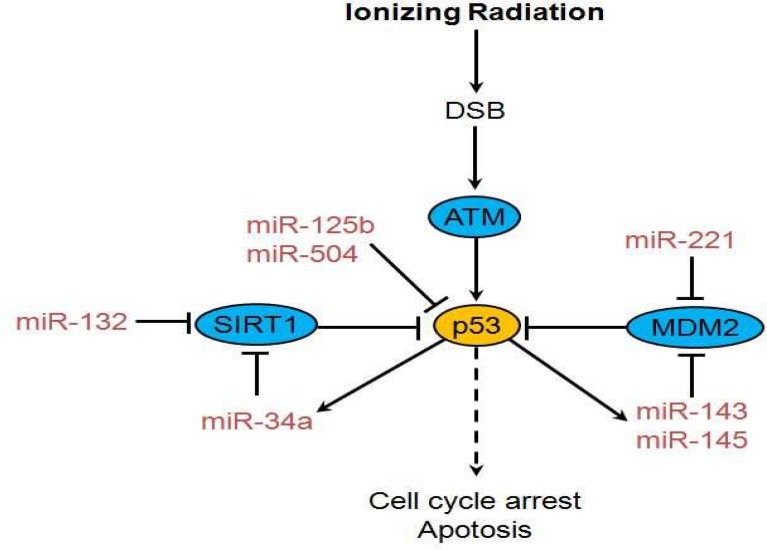
A simplified working model for p53-associated miRNAs. See text for more information (37-42)

**Fig 4:**
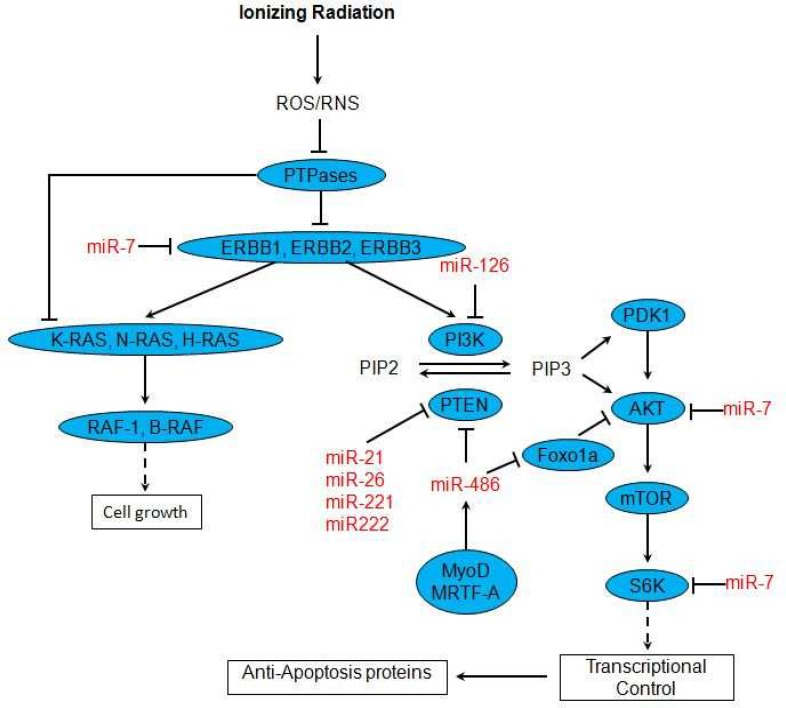
A simplified working model for role of miRNAs in radiation induced signal transduction pathways. See text for more explanation (43-54).

## Conclusion

Although it is not long time from the discovery of miRNAs, it have been cleared that these molecules have some roles in several cellular processes such as stress response, apoptosis, cellular differentiation and proliferation, cellular response to ionizing radiation and also these molecules have aberrant expression in a variety of diseases (55, 56). The main effect of IR on cells is DNA damage that results to cell cycle arrest or cell death especially in dividing cells. IR can also induce proliferation in the surviving fraction of cells. It has been discovered that miRNAs have fundamental role in several IR induced cell signaling events. In this review some miRNAs involved in cellular response to ionizing radiation have been discussed briefly; However, there are still many questions to be addressed. With thorough knowledge about the role of miRNAs in radiation response, it will be possible to design new strategies for improving radiotherapy efficiency and ultimately cancer treatment. 
